# Propensity of a picornavirus polymerase to slip on potyvirus-derived transcriptional slippage sites

**DOI:** 10.1099/jgv.0.001189

**Published:** 2018-12-03

**Authors:** Hazel Stewart, Allan Olspert, Benjamin G. Butt, Andrew E. Firth

**Affiliations:** 1 Division of Virology, Department of Pathology, University of Cambridge, Cambridge, UK; 2 School of Science, Department of Chemistry and Biotechnology, Tallinn University of Technology, Tallinn, Estonia

**Keywords:** picornavirus, RNA polymerase, transcription, potyvirus

## Abstract

The substitution rates of viral polymerases have been studied extensively. However less is known about the tendency of these enzymes to ‘slip’ during RNA synthesis to produce progeny RNAs with nucleotide insertions or deletions. We recently described the functional utilization of programmed polymerase slippage in the family *Potyviridae*. This slippage results in either an insertion or a substitution, depending on whether the RNA duplex realigns following the insertion. In this study we investigated whether this phenomenon is a conserved feature of superfamily I viral RdRps, by inserting a range of potyvirus-derived slip-prone sequences into a picornavirus, Theiler’s murine encephalomyelitis virus (TMEV). Deep-sequencing analysis of viral transcripts indicates that the TMEV polymerase ‘slips’ at the sequences U_6–7_ and A_6–7_ to insert additional nucleotides. Such sequences are under-represented within picornaviral genomes, suggesting that slip-prone sequences create a fitness cost. Nonetheless, the TMEV insertional and substitutional spectrum differed from that previously determined for the potyvirus polymerase.

In members of the negative-sense RNA virus families *Paramyxoviridae*, *Rhabdoviridae* and *Orthomyxoviridae*, the RNA-dependent RNA polymerase (RdRp) ‘stutters’ on short poly(U) tracts to polyadenylate the mRNAs [1]. Polymerase stuttering on poly(A) and poly(U) templates is also thought to maintain poly(A) tail length in picornaviruses [2, 3]. Polymerase stuttering or slippage can also occur within coding sequences to produce populations of transcripts with altered coding capacity, where nucleotide insertions or deletions allow access to alternative open reading frames. Where subject to purifying selection, this is known as ‘programmed polymerase slippage’ or ‘programmed transcriptional slippage’. Negative-sense RNA viruses in the taxa *Ebolavirus* and *Paramyxoviridae* have long been known to use polymerase slippage for gene expression [4, 5]. More recently, polymerase slippage was identified in the *Potyviridae*, the largest family of positive-sense RNA viruses of plants [6, 7]. In these viruses, polymerase slippage occurs at a highly conserved GAAAAAA (GA_6_) sequence, giving rise to transcripts with an additional adenylate. Translation of these transcripts allows expression of an essential ‘transframe’-encoded protein, P3N-PIPO. In a subgroup of potyviruses, slippage also occurs at a second GA_6_ site, enabling expression of the ‘transframe’-encoded protein, P1N-PISPO [8].

In potyviruses, the single nucleotide insertion rate varies between 0.8 and 2% (P3N-PIPO expression) [6–9] and 5 and 12% (P1N-PISPO expression) [7, 8], presumably influenced by flanking nucleotides. However, a significant number of substitution events are also observed, revealing a mechanism that has been termed ‘to–fro’ slippage, whereby the RdRp is hypothesized to undergo a ‘slip back, template, slip forward’ movement [10]. RNA duplex realignment following templating of the inserted nucleotide leads to the subsequent template nucleotide being ‘skipped’. The resulting transcript maintains the original protein-coding reading frame and length, but possesses a nucleotide substitution at the +7 position, 3′-adjacent to the GA_6_ slip site. When the reverse complement of the slippage site is present, substitutions are observed instead at the −1 position. The position of these substitutions allows determination of whether ‘to–fro’ slippage occurs during positive- or negative-sense synthesis. For the potyvirus polymerase, ‘to–fro’ slippage occurs mainly during synthesis of poly(A) rather than poly(U) regardless of the orientation of the GA_6_ sequence [10].

Given the evolutionary relatedness of the *Picornaviridae* and *Potyviridae* RdRps [11], it is reasonable to envision similar behaviour in both families. Consistent with this idea, bioinformatic analysis of both potyviral and picornaviral genomes revealed under-representation of A_*n*_ and U_*n*_ (*n*≥6) homopolymeric sequences when the functionally utilized potyviral slippage sites were excluded [6] (Fig. 1). Since deleterious effects of A_*n*_ and U_*n*_ sequences might also occur at the translational level as a result of ribosomal slippage, the picornavirus analysis was performed in all three reading frames. Selection against A_*n*_ and U_*n*_ sequences may reflect a propensity of the RdRp to slip at such sites, leading to a reduction in virus productivity from packaging of the defective transcripts. Slippage events may potentially also lead to more serious *in trans* antiviral effects, such as dominant negative interference by truncated versions of viral proteins and potential preferential MHC class I antigen presentation of slippage products [12–15].

In this study, we wished to investigate to what extent potyviral slippage sites lead to polymerase slippage in Theiler’s murine encephalomyelitis virus (TMEV), a model picornavirus in the genus *Cardiovirus*. As with other picornaviruses, TMEV has a polyadenylated positive-sense RNA genome of ~8 kB that encodes a polyprotein which is processed to produce the structural and non-structural viral proteins (Fig. 2a). The 5′ untranslated region (UTR) of ~1 kB contains an internal ribosome entry site. We used an infectious clone with sequence identical to GenBank Accession number X56019.1 except for three nucleotide differences, G2241A, A2390G and G4437A [16]. The wild-type (WT) sequence contains one A_6_ tract (in the region encoding 3C), one U_6_ tract (in the 5′ UTR) and no A_7_ or U_7_ tracts.

We inserted candidate polymerase slippage sites into the coding region, rather than the UTRs, so that insertions or deletions would lead to defective genomes that could not amplify without a helper virus. Indeed, as translation beyond the 2A region is required *in cis* for replication (at least for the related poliovirus) [17], such genomes would not be expected to replicate even in the presence of helper virus. To avoid altering the native viral proteins, we duplicated 24 amino acids of the 2A StopGo sequence via overlap PCR (TMEV-2SG; Fig. 2a) so that candidate slip-prone sequences could be inserted into restriction sites incorporated between the two StopGos. Translation of the StopGo sequence results in a peptide ending in NPGP that mediates co-translational polypeptide separation by preventing peptide bond formation between the glycine and final proline. Thus the inserted sequences would be co-translationally excised from the polyprotein with no effect on the amino acid sequences of the flanking 2A and 2B proteins.

Slippage mutant viruses were generated by the ligation of dsDNA linkers into a digested pTMEV-2SG backbone. Clones were designed based upon two potyviral slip-prone sequences (Fig. 2a). Following Olspert *et al*. [10], we use ‘TuMV’ to refer to sequences based on the turnip mosaic potyvirus P3N-PIPO slip site and ‘PISPO’ to refer to sequences based on the sweet potato feathery mottle virus P1N-PISPO slip site. Clones were made with the wild-type TuMV and PISPO GA_6_ slip sites (TuMV WT, PISPO WT), slip sites with an extra adenylate inserted (GA_7_) (TuMV+A) and their reverse complements (TuMV RC, PISPO RC, TuMV+A RC, PISPO+A RC). A PISPO+A mutant could not be rescued and was not used. Recombinant viruses were obtained from BHK21 cells transfected with T7 polymerase *in vitro* transcripts of the mutant plasmids. Following infection of naïve cells (MOI of 10), virus was harvested at multiple time points and titrated by plaque assay as previously described [16]. The mutant viruses did not exhibit significantly altered growth kinetics compared to wild-type TMEV (Fig. 2b).

To assess polymerase slippage, *in vitro*-transcribed RNA for each mutant viral genome was transfected into BHK21 cells in duplicate. Four hours post-transfection, cells were washed thoroughly and one replicate was frozen (sample ‘Cell T’, T denoting transfected). Supernatant was harvested from the remaining replicate at 24 h post-transfection (‘Virus 1’). The supernatant was used to infect naïve cells at an MOI of 0.1, which were subsequently harvested at 24 h post-infection (‘Cell I’, I denoting infected) at which point an additional supernatant sample was harvested (‘Virus 2’) (Fig. 2c). RNA was extracted from all samples using Trizol (Invitrogen) according to the manufacturer’s instructions and precipitated with isopropanol. RNA was reverse-transcribed using SuperScript III reverse transcriptase (Invitrogen) at 48 °C for 30 min (primer: 5′ ttccttggcacccgagaattccaCATGATATCCTCTTACTGCGTG 3′; upper case denotes template-derived nucleotides whereas lower case denotes Illumina-specific adaptor). Seventeen cycles of PCR were conducted using Q5 High Fidelity polymerase (New England Biolabs). The primers included the sequences required for library de-multiplexing (antisense: 5′ xxxxxxttccttggcacccgagaattccaCATGATATCCTCTTAC TGCGTG 3′; sense: 5′ aatgatacggcgaccaccgagatctacacgttcagagttctacagtccgacgatcAATGAACCCAGGCCCTAC 3′; xxxxxx denotes multiplex tag nucleotides). PCR libraries were separated by Tris-Borate-EDTA (10 %) polyacrylamide gel electrophoresis, target fragments were excised from the gel and the DNA extracted. The purified libraries were sequenced using the NextSeq500 platform (Illumina). As described previously [10], reads were checked for quality, clipped for adaptor sequence and de-multiplexed using the FASTX Toolkit (Hannon lab). Reads containing Ns, overly short reads, obvious contaminating reads from other libraries (errors in indexing) and reads less abundant than 0.01% of the most abundant read were not included in the analysis. The number of reads obtained for each sample ranged from 874 008 to 1 904 817. Reads were subsequently analysed for insertions, deletions and substitutions using custom scripts utilizing BioPython.

The T7 polymerase is known to slip on poly(A) and poly(U) homopolymeric sequences [18]. At the TuMV WT GA_6_ slip site, T7 slippage was previously measured at ~2.8% [6] although we observed an insertion rate of only 1.0% in this dataset (Fig. 3a, top left panel). The ‘Cell T’ samples were used to assess the combined contribution of T7 slippage and potential slippage during library preparation and sequencing. By performing infections at an MOI of 0.1, we expected to purge any virus genomes that were defective as a result of slippage occurring during T7 transcription or during virus replication following transfection, so that the ‘Cell I’ and ‘Virus 2’ insertion and deletion data should reflect the viral RdRp slippage rates.

In this initial experiment, the ‘Cell T’ samples were harvested at four hours post-transfection. The ‘Cell T’ samples were therefore used to differentiate the mutational spectrum of T7 transcription and library preparation from that of the viral RdRp. At this early time point only minimal viral replication would have occurred; therefore these samples were assumed to mainly reflect the *in vitro* T7 transcripts. To validate this assumption, the experiment was repeated (using independently transcribed *in vitro* transcripts) where a sample of T7 RNA was sequenced prior to transfection, alongside the remaining three samples for each mutant virus. This second dataset exhibited markedly similar results to the first, thus supporting the previous results (Fig. 3b).

For the four reverse complement mutants (i.e. those containing U_6_C or U_7_C in the positive-sense), the ‘Virus 1’, ‘Cell I’ and ‘Virus 2’ samples had a 2.2- to 3.1-fold increase in single-nucleotide insertions compared to the input, reaching levels of 0.71±0.16 and 0.76±0.16% for the TuMV RC and PISPO RC mutants, and 4.2±0.5 and 3.7±0.5% for the TuMV+A RC and PISPO+A RC mutants, respectively (Fig. 3a, top panel). The values show means±standard deviaations based on six virus and/or two input samples combined over panels A and B of Fig. 3. These results contrast with the potyvirus RdRp, for which the TuMV RC and PISPO RC slippage rates were quite different from each other (0.53±0.04 and 2.1±0.34%, respectively). Translation, replication and packaging of picornaviral RNA are thought to be linked, leading to preferential *in cis* packaging of intact viral genomes [17, 19, 20]. However we observed similar levels of slippage transcripts in both supernatant (‘Virus 1’, ‘Virus 2’) and cell lysates (‘Cell I’). This can be reconciled with previous results by noting that a translationally intact genome may lead to formation of a replication vesicle containing that parental genome, but that this may produce a mixture of wild-type and slippage transcripts that may be packaged with equal efficiency as they emerge from the vesicle. For the other three mutants (GA_6_ and GA_7_ in the positive-sense), the ‘Virus 1’, ‘Cell I’ and ‘Virus 2’ samples had decreased levels of insertions compared to the input, indicating that the viral RdRp has a lower tendency to slip on these sequences than the T7 polymerase. Since slippage may also occur during library preparation, these values – which ranged through 0.50±0.12% (TuMV WT), 1.1±0.16% (PISPO WT) and 1.6±0.24% (TuMV+A) – should be considered as upper bounds on the viral RdRp slippage rates.

We also quantified deletional slippage (Fig. 3a, second panel). TuMV+A RC and PISPO+A RC both exhibited high levels of presumably T7-derived deletional slippage (4.8±0.71 and 6.5±1.0 %, respectively); significant rates of slippage on similar U_*n*_ tracts (*n*>6) by T7 polymerase have been noted previously and are an essential component of efficient transcription termination [18, 21]. This was purged to levels of 0.35±0.15 and 0.23±0.19% following virus replication. The TuMV+A mutant had similar slippage both in the input (1.8±0.11 %) and following virus replication (1.5±0.26 %), suggesting that both the T7 and viral polymerases had similar deletional slippage propensities at this site. Only low levels of deletional slippage (upper bounds <0.14 %) were observed during replication of GA_6_ or U_6_C slip-site viruses.

Following Olspert *et al*. [10], we inspected reads for evidence of ‘to–fro’ slippage – that is, a substitution to A or U immediately following or immediately preceding an A_*n*_ or U_*n*_ slip site, respectively (red bars, Fig. 3). Whereas virus infection at low MOI would be expected to purge insertion/deletion mutations, substitutions within the inter-StopGo insert region would likely not be subject to strong selective pressure. Thus substitutions introduced by the T7 polymerase, or during the course of virus growth, would likely be retained and propagated.

At position +7 (i.e. 3′-adjacent to the slip site) of the U_6–7_C slip sites, there appeared to be a component of C-to-U substitutions (Fig. 3a, third panel, RC mutants, red bars) which was clearly not derived from the input RNA. In contrast, levels of C to not-U substitutions (grey bars) were similar between virus-derived and input RNA. These results indicate that ‘to-fro’ slippage by the viral RdRp occurs at U_6–7C_ slip sites during positive-sense synthesis. In contrast, for the GA_6–7_ slip sites, levels of ‘to A’ substitutions at position +7 were similar between virus-derived and input RNA, indicating that ‘to-fro’ slippage does not occur to appreciable levels during positive-sense synthesis at GA_6–7_ slip sites (Fig. 3, 3rd panel, non-RC mutants, red bars). At the −1 position (i.e. 5′-adjacent to the slip site) differences between input and virus-derived RNA were less striking, although the four TuMV mutant viruses showed a possible increase in G to A (TuMV WT, TuMV+A) and C to U (TuMV RC, TuMV+-A RC) substitutions compared to input (Fig. 3, 4th panel, red bars).

Due to the background of spurious mutations, Olspert *et al*. focussed on sites with potyvirus RdRp substitution levels ≥0.5% [10]. For the TMEV RdRp, we only observed virus-specific substitutions approaching this level for the TuMV+A RC site (Fig. 3a, third panel). For the TuMV WT slip site (GA_6_), G-to-A substitutions at position +7 were 36-fold lower than previously observed with the potyvirus RdRp (0.015±0.003 versus 0.54±0.07%); and for the TuMV RC slip site (U_6_C), C-to-U substitutions at position −1 were 33-fold lower than with the potyvirus RdRp (0.065±0.014 versus 2.2±0.5%) [10].

To summarize, our data indicate that the TMEV RdRp permits insertional slippage at levels of 0.46–4.3% on A_6–7_ and U_6–7_ sequences, with higher levels of slippage occurring when poly(U) is present in the positive strand. The highest levels of ‘to–fro’ slippage occur at position +7 for the TuMV+A RC slip site, suggesting that slippage on U_6–7_ sequences may occur predominantly during positive-sense synthesis. Picornaviral RdRps are thought to use RdRp stuttering to maintain genomic poly(A) tail length during replication [2], where the positive-sense sequence is poly(A) rather than poly(U), though slippage may occur during synthesis of either strand, contributing to the final poly(A) tail length. Surrounding RNA structures may contribute to the efficiency of this event; for example, a cis-element of enteroviruses (located within the 3′ UTR) is thought to enhance polymerase slippage on the negative-sense poly(U) template to facilitate polyadenylation of the positive-sense viral genome during replication [22]. It is therefore likely that slippage propensity will differ between sites as a result of flanking sequences and/or homopolymer length; however, our study was specifically aimed at potyviral-like slip sites.

It remains possible that picornavirus polymerases may have evolved an increased propensity for slippage in specific genera or species, or that polymerase slippage may be utilized on specific sequences that differ from the potyvirus-derived sequences tested herein. For example, encephalomyocarditis virus (also in the *Cardiovirus* genus) has a long poly(C) tract in its 5′ UTR that is associated with heightened virulence [23]. However there is no direct evidence that polymerase slippage is used during evolution of the length of this tract (a possible alternative is recombination). Our bioinformatic analysis did not reveal any significant under-representation of poly(C) tracts in picornavirus coding regions which suggests that – at least for short, N_6-7_ tracts – they are less prone to spurious slippage events than poly(A) or poly(U).

Although both potyviruses and cardioviruses possess superfamily I RdRps [11], the tendency of each RdRp to slip upon particular nucleotide sequences appears distinct, with the potyvirus RdRp preferentially slipping during synthesis of GA_6_ regardless of sense. Whereas potyviruses utilize polymerase slippage to access novel ORFs, there are no known cases of this occurring in picornaviruses. As mentioned above, non-programmed polymerase slippage within coding sequences results in defective transcripts which may lead to various negative effects [12–15], and these factors may contribute to the significant under-representation of U_6–7_ and A_6–7_ sequences within picornaviral genomes. This research contributes to our understanding of the mechanisms that shape RNA virus genomic diversity and highlights differences between related viral polymerases, where the potyvirus RdRp may have co-evolved with the expression of the essential P3N-PIPO protein to be specifically tuned to facilitate slippage on the GA_6_ slip sites that potyviruses functionally use for gene expression.

## Figures and Tables

**Fig. 1:**
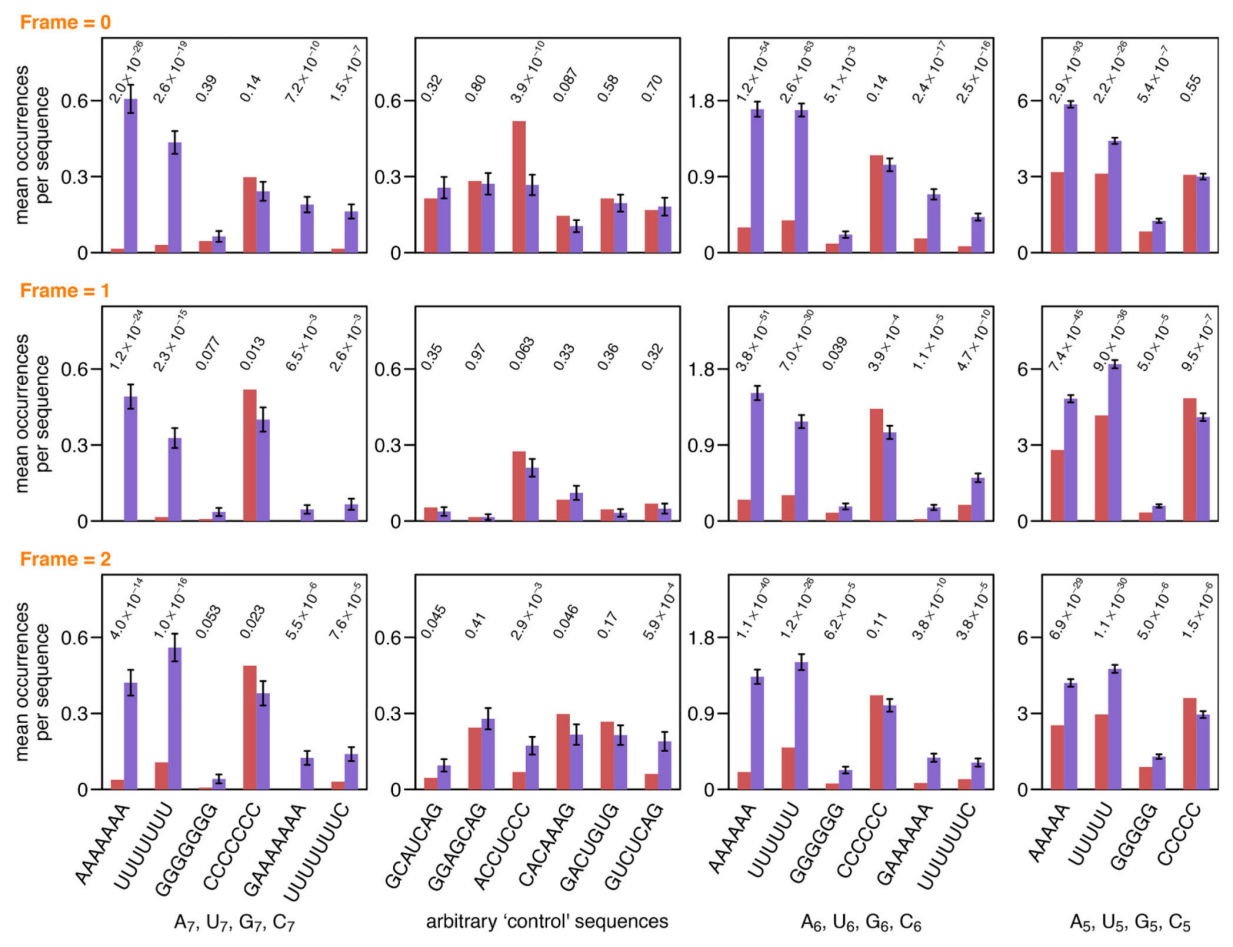
Poly(A) and poly(U) sequences are under-represented in picornavirus genomes. An analysis of 131 *Picornaviridae* NCBI RefSeqs indicates strong selection against A_7_, U_7_, A_6_ and U_6_ sequences. A_5, 5_, poly(C), poly(G) and several arbitrary heptanucleotides are included for comparison. Red bars indicate the mean observed frequency per polyprotein ORF of the indicated sequences. Polyprotein ORFs were also randomly shuffled 1000 times while maintaining amino acid sequence and codon bias (as previously described [6] except that here each of the three reading frames was analysed separately). Purple bars indicate mean frequencies per polyprotein ORF in the shuffled sequences. Error bars indicate standard deviations. Values above bars indicate two-tailed *z*-test *p*-values showing that the observed counts are statistically different from the expected counts.

**Fig. 2:**
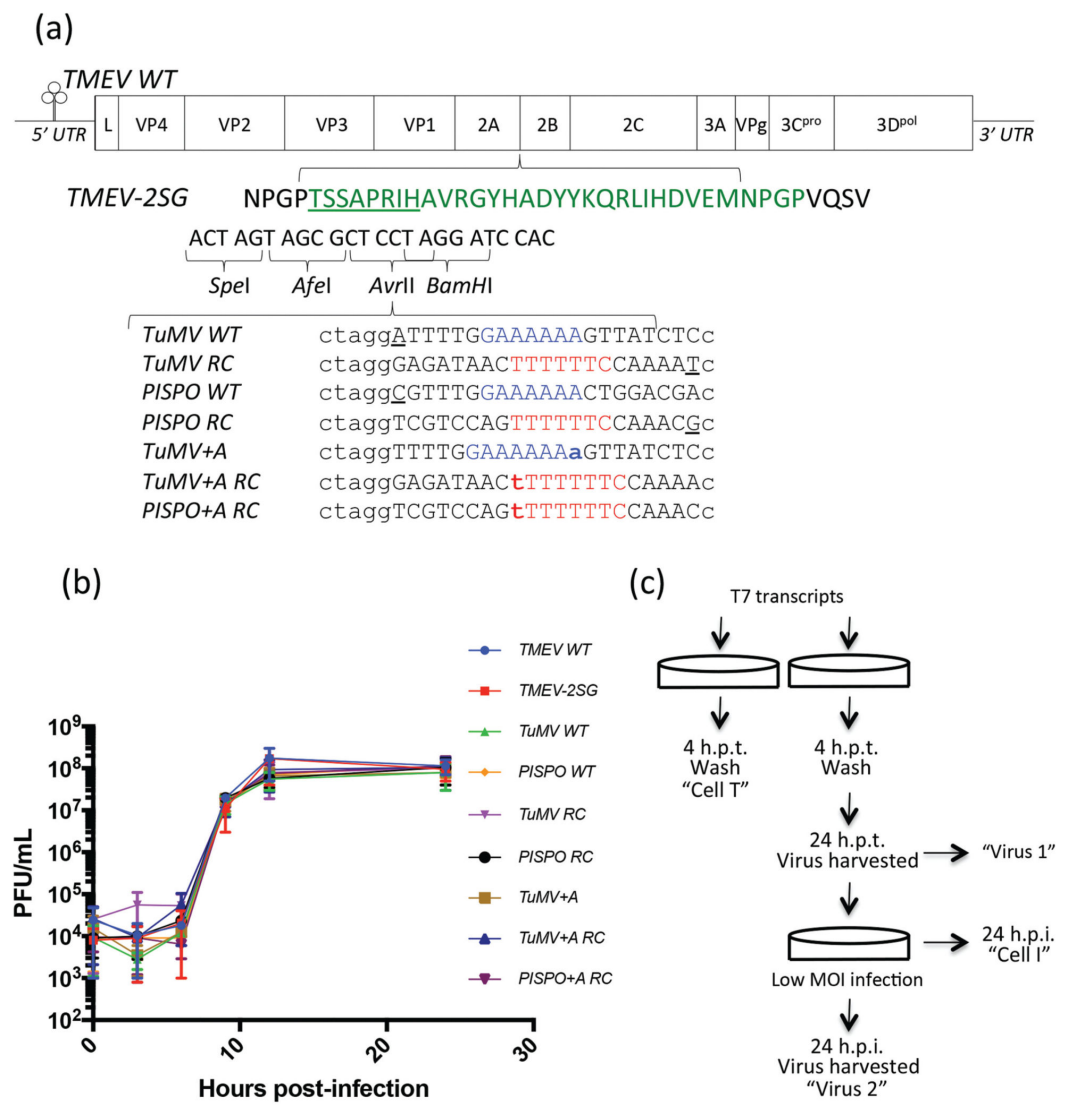
Introduction of duplicate StopGo and potyvirus-derived slip-site sequences do not affect TMEV growth kinetics. (a) Schematic of the TMEV WT genome, indicating the duplicate 2*A* StopGo peptide (green) inserted to create TMEV-2SG. Underlined residues indicate those encoding cloning sites (detailed underneath) that do not contribute to StopGo function. Wild-type flanking residues are shown in black. Seven sequences based on potyvirus polymerase slippage sites were inserted into TMEV-2SG. Slip sites are shown in blue (native potyviral slip sites and ‘+A’ variants) or red (reverse complements); additional nucleotides extending the slip site are indicated in bold; other sequences have an additional nucleotide (underlined) added at the end of the insert to maintain the same reading frame (this is checked in each read to guard against inter-sample contamination from the +A mutants as a result of multiplex tag misassignments). Upper case indicates nucleotides derived from potyvirus genomic sequences; lower case indicates flanking nucleotides used for cloning. (b) One-step growth curves (*n*=2) of mutant viruses indicate they do not exhibit significantly altered growth kinetics compared to either TMEV WT or TMEV-2SG. (c) Schematic of the experimental protocol followed to obtain the initially transfected cells (‘Cell T’), first-round virus (‘Virus 1’), infected cells (‘Cell I’) and second-round virus (‘Virus 2’) samples.

**Fig. 3:**
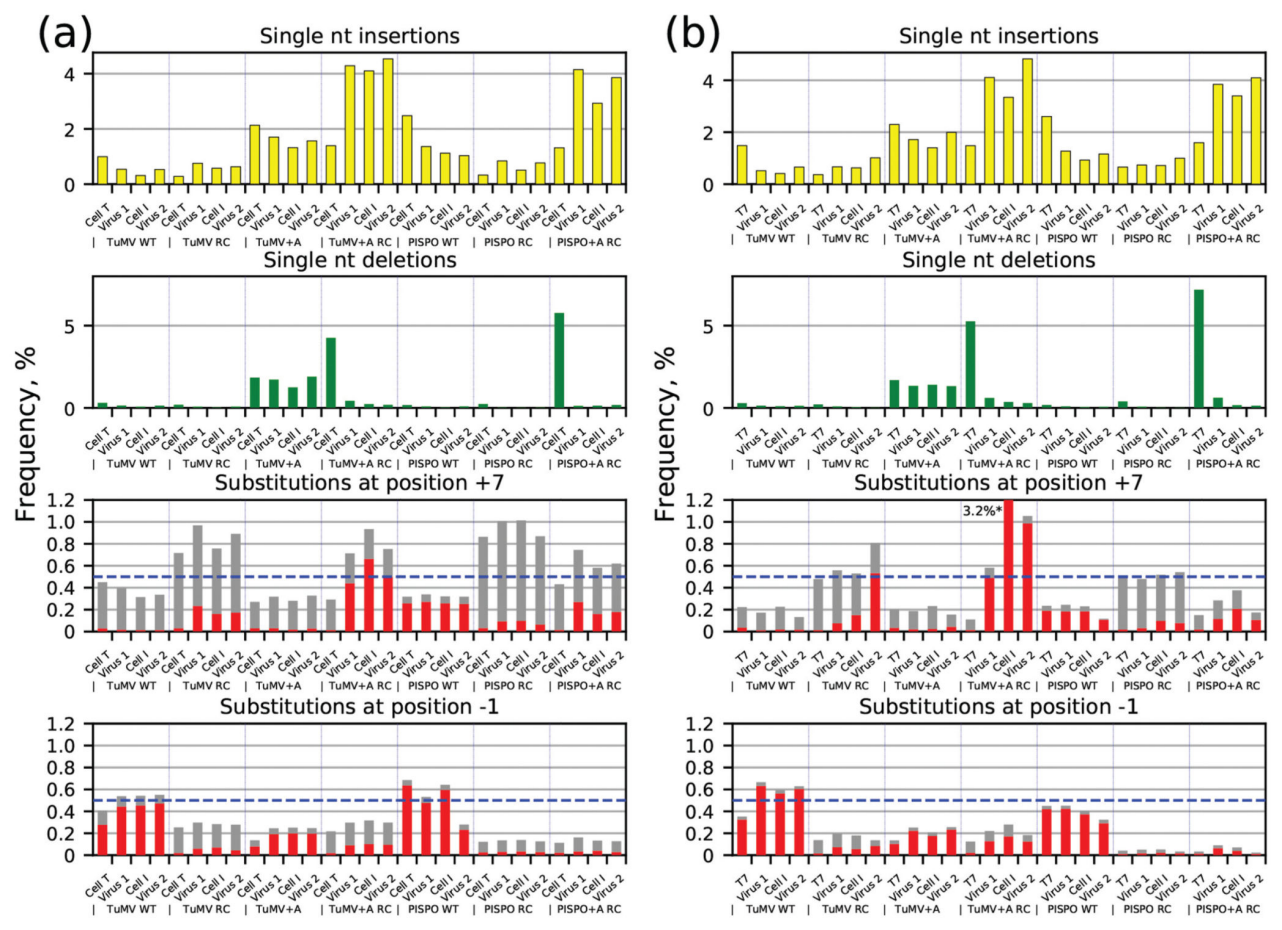
Polymerase slippage occurs preferentially when poly(U) tracts are present within the cardiovirus positive strand. RNA was extracted from virus-containing supernatant from transfected (Virus 1) or infected (Virus 2) cells or infected intact cells (Cell I). Duplicate transfected cells were harvested 4 h post-transfection (Cell T) (a), or a sample of the input T7-derived RNA was processed (T7) (b). RNA was deep-sequenced across the slip-site region and the percentages of reads containing single-nucleotide insertions (yellow), deletions (green) or substitutions were assessed. Substitution rates to A (TuMV WT, PISPO WT and TuMV+A samples) or U (TuMV RC, PISPO RC, TuMV+A RC and PISPO+A RC samples) are shown in red; substitution rates to other nucleotides at the same positions are plotted above in grey. The blue dashed line corresponds to 0.5 %. Note for the +A mutants, the substitution rates are calculated at position +8 instead of +7 due to the extra nucleotide inserted in the slip site. Values that extend beyond the *y*-axis limit are marked with an asterisk, with the value listed alongside.

## Abbreviations

p.i: post infection
RdRp: RNA dependent RNA polymerase
TMEV: Theiler’s murine encephalomyelitis virus
UTR: untranslated region
